# Alternations in Dynamic and Static Functional Connectivity Density in Chronic Smokers

**DOI:** 10.3389/fpsyt.2022.843254

**Published:** 2022-04-21

**Authors:** Zhengui Yang, Mengmeng Wen, Yarui Wei, Huiyu Huang, Ruiping Zheng, Weijian Wang, Xinyu Gao, Mengzhe Zhang, Jingliang Cheng, Shaoqiang Han, Yong Zhang

**Affiliations:** ^1^Department of Magnetic Resonance Imaging, The First Affiliated Hospital of Zhengzhou University, Zhengzhou, China; ^2^Key Laboratory for Functional Magnetic Resonance Imaging and Molecular Imaging of Henan Province, Zhengzhou, China; ^3^Engineering Technology Research Center for Detection and Application of Brain Function of Henan Province, Zhengzhou, China; ^4^Engineering Research Center of Medical Imaging Intelligent Diagnosis and Treatment of Henan Province, Zhengzhou, China; ^5^Key Laboratory of Magnetic Resonance and Brain Function of Henan Province, Zhengzhou, China; ^6^Key Laboratory of Brain Function and Cognitive Magnetic Resonance Imaging of Zhengzhou, Zhengzhou, China; ^7^Key Laboratory of Imaging Intelligence Research Medicine of Henan Province, Zhengzhou, China

**Keywords:** functional connectivity density, static, dynamic, chronic smokers, fMRI, addiction

## Abstract

Previous studies have implicated abnormal functional coordination in brain regions of smokers. Neuroimaging studies demonstrated alternations in brain connectivity by using the resting-state functional connectivity (rsFC) method which arbitrarily chooses specific networks or seed regions as priori selections and cannot provide a full picture of the FC changes in chronic smokers. The aim of this study was to investigate the whole-brain functional coordination measured by functional connectivity density (FCD). As the variance of brain activity, dynamic FCD (dFCD) was performed to investigate dynamic changes of whole-brain integration in chronic smokers. In total, 120 chronic smokers and 56 nonsmokers were recruited, and static FCD and dFCD were performed to investigate aberrance of whole-brain functional coordination. Shared aberrance in visual areas has been found in both static and dFCD study in chronic smokers. Furthermore, the results exhibited that both heavy and light smokers demonstrated decreased dFCD in the visual cortex and left precuneus, and also increased dFCD in the right orbitofrontal cortex, left caudate, right putamen, and left thalamus compared with nonsmokers. In addition, alternations of dFCD have been found between heavy and light smokers. Furthermore, the dFCD variations showed significant positive correlation with smoking-related behaviors. The results demonstrated that chronic smokers not only have some initial areas, but also have some regions associated with severity of cigarette smoking. Lastly, dFCD could provide more subtle variations in chronic smokers, and the combination of static and dFCD may deepen our understanding of the brain alternations in chronic smokers.

## Introduction

Cigarette smoking is considered as the leading cause of preventable disease in the world. It has a negative influence on health, economic and society. Nearly 6 million deaths and over a half trillion dollars in healthcare costs in the world are attributed to smoking (1). In addition, cigarette smoking has also been associated with the higher risk of cognitive decline and dementia (2, 3). According to previous studies, chronic smokers lose at least 10 years of life compared to nonsmokers (4). Although a large number of smokers are willing to quit smoking, only a few people could succeed without the help of medication or other treatment (5). In fact, most of them relapse within only 1 week (6). Therefore, a better understanding of the neural effects of smoking in human brains is important to help chronic smokers quit smoking. There exists an amount of evidence stating that that cigarette smoking has a negative influence on functional alternations in the brain. For instance, substance addiction might alter the sensitivity of brain regions, including motivation and reward (7).

Numerous neuroimaging studies have been conducted to explore alternations of functional coordination in several brain regions and networks of smokers in recent years. Resting-state functional magnetic resonance imaging (fMRI) studies have reported that chronic smokers showed widespread abnormal functional connectivity (FC) in some brain regions. The orbitofrontal cortex (OFC) is thought to integrate and modulate activity from several limbic areas involved in reward processing (8). Activation has been recorded in brain regions including the caudate, OFC, and parahippocampal gyrus during control scanning in response to smoking-related images (9). Compared with nonsmokers, smokers had lower connectivity associated with key network hubs, including the default mode network (DMN) (10). Lower FC has been found between the caudate and OFC in smokers (11). Decreased FC in the left thalamo-precuneus has also been found in relapsing addicts (12). Furthermore, widespread FC attenuation has been observed in the reward circuit of smokers compared with non-smokers (13). In addition, neuroimaging studies have found alternations in brain coordination among different severity of smoking. That is, smokers with greater nicotine dependence severity tend to demonstrate greater engagement of sensorimotor and motor preparation circuits, and Fagerström Test for Nicotine Dependence (FTND) scores were positively associated with increased connectivity between insular and dorsal striatum and early visual processing cortex (14). Therefore, this research attempted to identify the alternations of brain coordination between smokers with different nicotine dependence severity by using a cross-sectional sample. However, all above FC studies required prior assumption and cannot provide a landscape of whole-brain FC changes, which might exist some limitations for exploratory analyses.

Recently, resting-state FC density (FCD) has been performed to measure the number of resting-state functional connections of a given voxel with all other voxels in the whole brain (15). This has been generally used in some psychiatric disorders to investigate the aberrance in brain static FC (16, 17). Unlike the seed-based FC method, FCD is a kind of method that is defined by the functional connections between each voxel in the brain. It does not need previous hypothesis (15). Therefore, FCD might be an approach that could provide more information compared with FC. Higher FCD of a specific voxel indicates that it is functionally connected with a large number of voxels within the brain and that the voxel plays a more important role in information processing compared with others. A previous study has used this method to demonstrate brain coordination in smokers of different states, i.e., abstinence and satiety state (16). Considering the dynamic nature of brain activity (18), the sliding window correlation approach has been widely used in the FC method to demonstrate the collaboration of brain regions by measuring the time-varying covariance of their neural signals during resting-state (19). The aberrance of the variance of FC has been conducted in many other mental diseases such as depression and schizophrenia (20, 21). In addition, the dynamic FCD (dFCD) method has been conducted in some diseases, including generalized anxiety disorder (GAD) and benign epilepsy with centrotemporal spikes (22, 23), despite not yet being performed on smokers in previous research. In the current study, the sliding window correlation approach was combined with FCD to evaluate the variance of brain activity. In conclusion, the static FCD provides a new avenue to illustrate the FC of whole brain, whereas the dFCD was calculated to identify the variance of brain activity by dividing the whole time series into different segmentations. Therefore, exploiting the methods of static and dFCD could help to provide supplementary evidence to uncover the aberrance of brain areas between chronic smokers and nonsmokers.

In the current study, we aimed to identify the aberrance of brain FC caused by cigarette smoking using static and dFCD method in 120 chronic smokers and 56 age- and gender- matched nonsmokers. Based on previous studies, we hypothesized that (1) the static and dFCD method could reveal shared and different brain areas showing functional abnormalities (20), and that (2) there might exist some brain regions that can be associated with the severity of smoking. In addition, the correlation analyses were performed to identify the relationships between FCD measurements and smoking-related behaviors.

## Materials and Methods

### Participants

In total, 120 chronic smokers and 56 nonsmokers were recruited from online advertisement, and all the participants are males. Then, 120 chronic smokers were divided into 61 nonsmokers (cigarette per day>20) and 59 light smokers (cigarette per day <20) (24). Smokers eligible for the study included those that: met the DSM-IV criteria for nicotine dependence, smoked at least 10 cigarettes per day for the past 5 years, had no period of smoking abstinence longer than 3 months in the past years, and whose smokers' nicotine addiction was assessed by the FTND. Nonsmokers were those who smoked <5 cigarettes in his lifetime. The exclusion criteria included physical illnesses, such as brain tumor, obstructive lung disease; a history of neurological and psychiatric diseases; addiction to other substances (except nicotine); and those with contraindications to MRI. This study aimed to study differences in neural activity between smokers in satiety state and nonsmokers. Smokers were required to smoke a cigarette 30-45 min before examination to prevent withdrawal symptoms. All the participants provided informed consents to the study protocol. The study was reviewed and approved by the Local Medical Ethics Committee of the First Affiliated Hospital of Zhengzhou University.

### Data Acquisition

Magnetic resonance imaging data were obtained using a 3.0T German Siemens Magnetom Skyra magnetic resonance imaging equipment with a sixteen-channel prototype quadrature birdcage head coil. Participants were instructed to keep their eyes closed, not to fall asleep, and to maintain their head motionless during scanning. Functional images were obtained using an echo planar imaging sequence with the following parameters: repetition time (TR)/echo time = 2,000/30 ms, matrix size = 64 × 64, flip angle = 80°, field of view = 240 × 240 mm, voxel size = 3 × 3 × 3 mm, slices = 36, slice thickness = 4 mm, no gap, and a total of 180 volumes.

### Image Analysis and Preprocessing

The Data Processing and Analysis of Brain Imaging (DPABI v3.0) (http://rfmri.org/DPABI) toolbox was used to preprocess the functional imaging data. Imaging preprocess was performed as follows. The first 5 volumes from each subject were discarded. Then, functional images were slice-timing corrected, realigned (cut off <2.5 mm or 2.5°), spatially normalized to the Montreal Neurologic Institute space, and re-sampled to 3 × 3 × 3 mm^3^. Next, several spurious variances (24 head motion parameters, global signals, ventricular signals, and white matter signals) were regressed using multiple linear regression analysis. For a precise head motion correction, the parameters from scrubbing data were also regressed. Previous researches reported that the global signal regression could improve the accuracy of FC calculation (25). Thus, we regressed the global signal in our study. Framewise displacement (FD) was calculated for each time point (26), and participants with mean FD value exceeding.5 mm were excluded. Subsequently, functional images were trended and temporal band-pass filtered.01 Hz~0.08 Hz.

### Estimation of Static and Dynamic FC Density

In comparison to static FCD, dFCD is a method combining with sliding window correlation approach looking at variation across the time series. To calculate the static FCD, Pearson's linear correlation was used to evaluate the strength of the FC between voxels. Two voxels with a correlation coefficient of R >0.6 were considered significantly connected. This threshold was proposed to be the optimal threshold for calculating resting-state FCD in a previous study (15). The dFCD analysis was performed by using Dynamic Brain Connectome (DynamicBC) toolbox (27) (V2.0 http://restfmri.net/forum/DynamicBC). The window length is a key parameter in sliding window correlation calculation. According to the rule of thumb, the minimum window length should exceed 1/f_min_, where f_min_ denoted the minimum frequency of time courses (28). Therefore, we selected 50 TRs as a window wise and a window overlap of 90%. To certify the robustness of the sliding-window analysis, we also examined other window wises that were included in validation analysis. In each sliding window, we obtained a global FCD map in each window by computing Pearson's correlations between the voxels within the whole brain. Two voxels were considered to be connected when the Pearson's correlation coefficient of the two voxels was greater than a given threshold *r* = 0.2 according to the significant level of *p* < 0.001 (uncorrected) in order to eliminate weak correlation which may be caused by noise (29). The temporal variability was calculated by the SD of FCD across sliding windows. Then, the temporal variability map of each subject was normalized into a z-score matrix. Subsequently, all the normalized images were smoothed (6 × 6 × 6 mm full width at half maximum Gaussian kernel).

### Statistical Analysis

One-way ANOVA was conducted among the three groups for voxels within the whole brain to explore the alternation of static and dFCD among nonsmokers, light smokers, and heavy smokers. In this step, age, years of education, and mean FD were included as covariates. The threshold of gaussian random field correction (GRF) was performed on the F-value map with voxel *p* < 0.005, cluster *p* < 0.01 (two-tailed).

Then, to investigate the details about the aberrance among these three groups, a two tailed two-sample *t*-test was performed between each pair of groups based on those brain regions having a significant F value alternation among three groups to detect the between-group differences in static and dFCD. Region of interest (ROIs) were defined as spheres with radius of 6 mm centered at the MNI coordinate reported for the brain regions having a significant F value. To examine the association between the abnormalities of FCD measurements and cigarette smoking, correlation analyses were performed between FCD measurements and smoking-related behaviors including pack-years and FTND.

### Validation Analyses

Since there is no clear conclusion on the optimal window length for the sliding window method. We validated our results with window lengths of 30 and 80 TRs. The additional window lengths were 30 and 80 TRs. In addition, we have conducted analyses that global signal was not regressed to verify the stability of our results. The corresponding results are shown in the Supplementary Materials.

To exclude the effect of head motion on observed results, Pearson correlation was calculated between the dFCD of ROI signals with mean FD among three groups. In addition, the mean FD was compared among three groups and between heavy smokers and light smokers.

## Results

### Demographics and Clinical Characteristics

In total, there were 61 heavy smokers, 59 light smokers, and 56 nonsmokers included in the current study. No significant difference was found among groups in terms of sociodemographic characteristics, such as age and year of education. The detailed demographic information and smoking behaviors were shown in Table 1.

**Table 1:** Demographic and smoking behaviors.

	**Heavy smokers**	**Light smokers**	**Nonsmokers**	***p* value**
Sex (male/female)	61/0	59/0	56/0	–
Age (mean±SD)	36.78 ± 7.78	35.40 ± 8.96	36.52 ± 7.48	0.096^a^
Education (mean±SD)	14.23 ± 2.35	14.56 ± 2.51	15.23 ± 3.09	0.084^a^
FTND (mean±SD)	5.39 ± 1.99	2.29 ± 1.57	–	–
Cigarette per day (mean±SD)	26.89 ± 6.88	12.02 ± 4.15	–	–
Peak-year (mean±SD)	26.89 ± 13.33	9.96 ± 7.04	–	–

*FTND, Fagerström Test for Nicotine Dependence; Pack-years, (Years of smoking × Cigarettes smoked per day)/20.*

a *One-way ANOVA*.

### Static FCD Between Chronic Smokers and Nonsmokers

**Figure 1:**
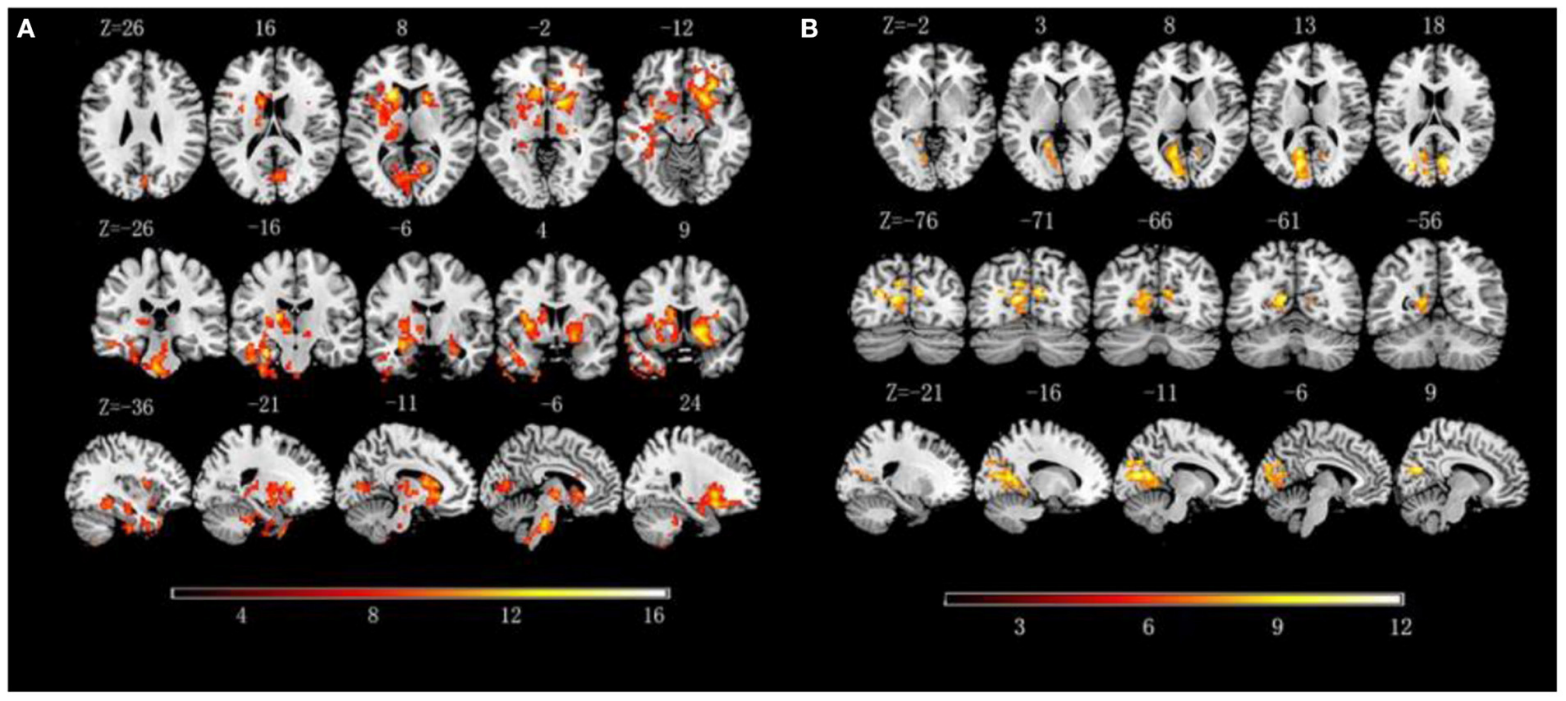
**(A)** Significant alternations of dynamic functional connectivity density (dFCD) among three groups; **(B)** Significant alternations of static functional connectivity density (FCD) among three groups.

The three groups presented significantly static FCD of brain regions in the visual cortex, including the bilateral calcarine and right cuneus (GRF corrected *p* < 0.005, *F* = 6.20, Table 2, Figure 1).

**Table 2:** Brain regions with changed static and dynamic functional connectivity density (FCD) among the three groups.

**Indices**	**Cluster**	**Voxels**	**Brain region**	**Sphere**	**Peak intensity**	**MNI coordinate**
Dynamic FCD:	1	1671	Caudate	L	16.79	−18, 18, 12
			ParaHippocampal Gyrus	L	16.53	−30, −18, −24
			Thalamus	L	12.13	9, −12, 9
	2	636	Frontal Gyrus	R	14.32	21, 12, −12
			Pallidum	R	14.25	15, 9, −3
			Orbitofrontal Cortex	R	13.89	30, 27, −12
	3	74	Superior Temporal Gyrus	L	11.43	−69, −21, 6
			Middle Temporal Gyrus	L	11.02	−69, −33, 3
	4	269	Cuneus	R	10.99	3, −72, 21
			Calcarine	R	10.90	15, −63, 9
			Calcarine	L	9.21	−12, −75, 9
	5	60	Thalamus	R	9.512	9, −12, 9
	6	78	Precuneus	L	9.61	−6, −48, 54
Static FCD:	1	380	Occipital Cortex	L	12.19	−27, −75, 18
			Calcarine	L	11.47	−12, −75, 12
	2	72	Calcarine	R	10.36	9, −72, 18
			Cuneus	R	6.68	12, −87, 15

*GRF corrected, voxel p <0.005, cluster p <0.01; L, left; R, right*.

The *post-hoc* results demonstrated that both heavy and light smokers showed decreased static FCD in the bilateral calcarine, and heavy smokers showed significantly lower static FCD in the right calcarine compared with light smokers. As for right cuneus, only heavy smokers showed significantly decreased static FCD in comparison to nonsmokers.

### Dynamic FCD Differences Between Chronic Smokers and Nonsmokers

The three groups presented significantly different dFCD of brain regions in the right OFC, dorsal striatum (left caudate and right putamen), visual cortex (bilateral calcarine and right cuneus), DMN [left parahippocampal gyrus, left precuneus and middle temporal gyrus (MTG)], and bilateral thalamus (*p* < 0.005, GRF corrected, *F* = 6.20, Table 2, Figure 1).

**Figure 2:**
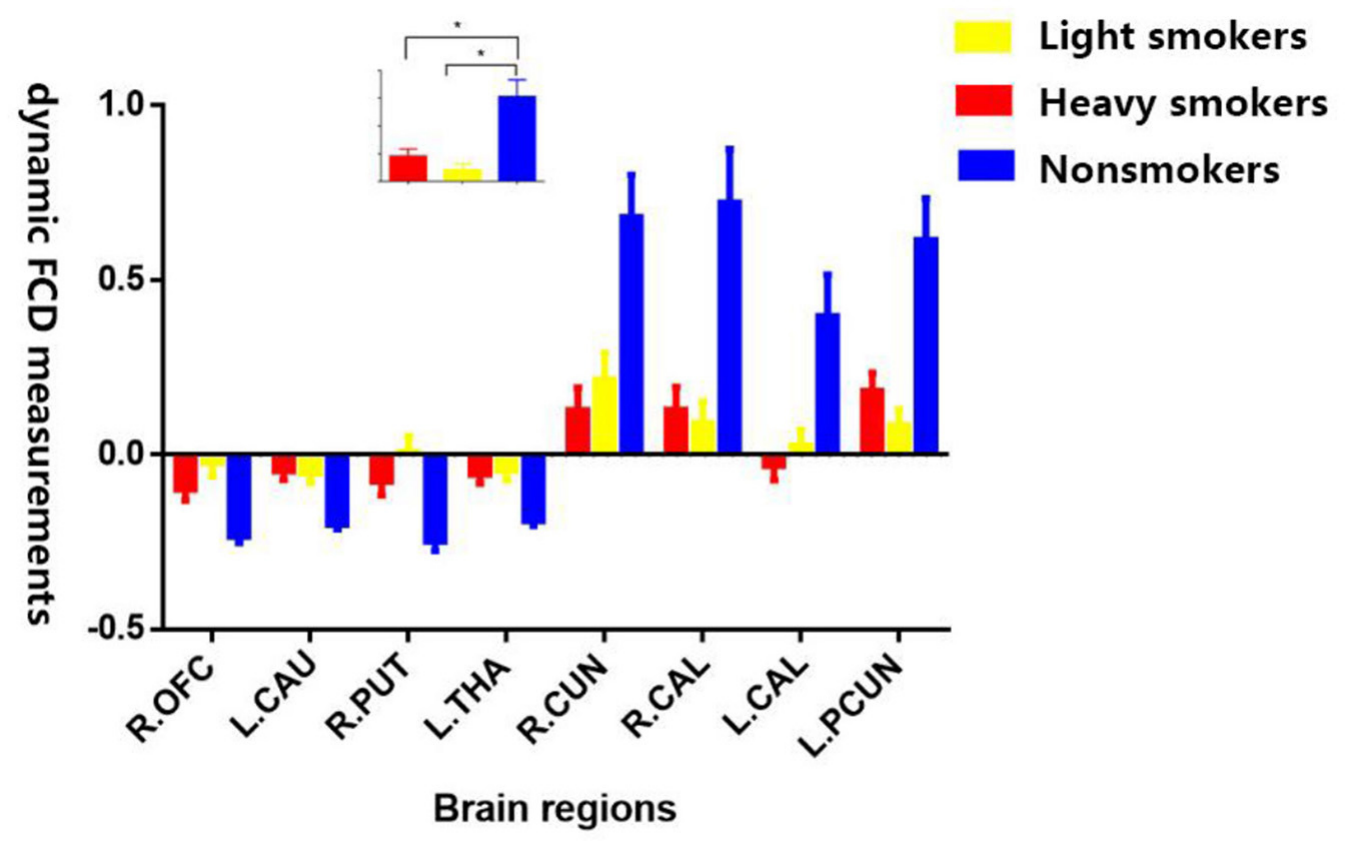
Shared alternations of dFCD measurements of brain regions in both heavy smokers and light smokers compared with nonsmokers. R.OFC, right orbitofrontal cortex; L.CAU, left caudate; R.PUT, right putamen; L.THA, left thalamus; R.CUN, right cuneus; R.CAL, right calcarine; L.CAL, left calcarine; L.PCUN, left precuneus. (The subplot means that both heavy and light smokers have significant alternations of dFCD measurements compared with nonsmokers). * means difference between the two groups has statistical significance.

**Figure 3:**
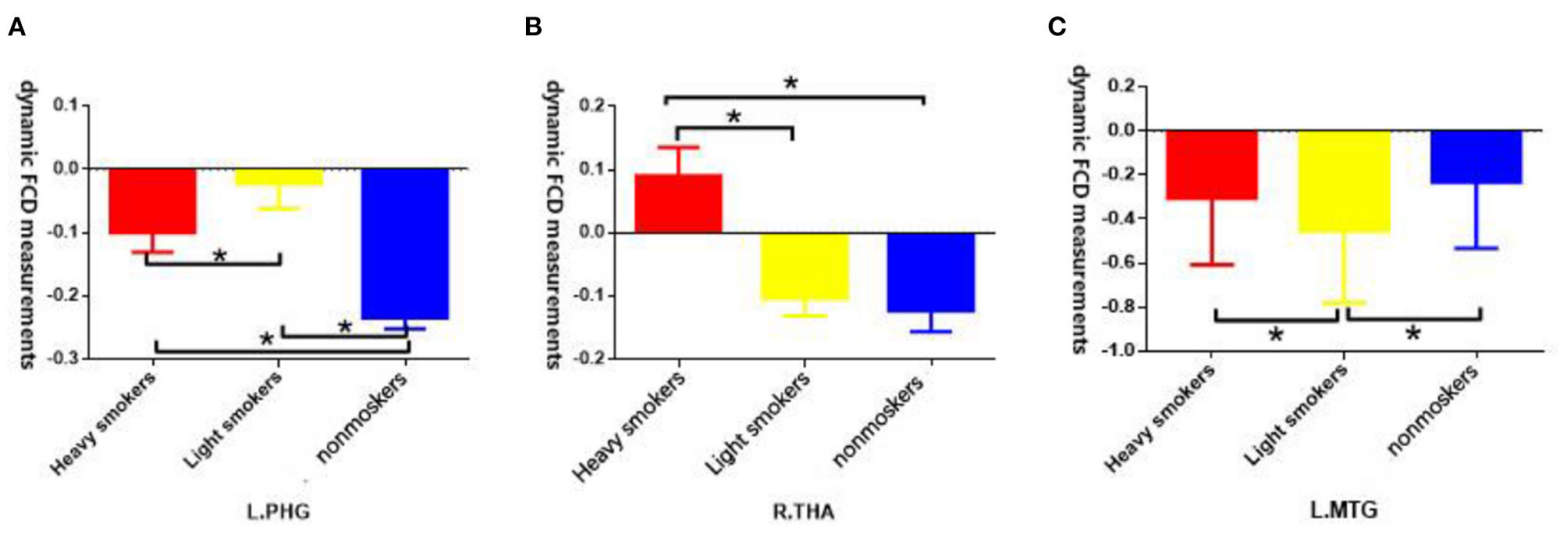
**(A)** Both heavy smokers and light smokers showed significant alternations of dFCD measurements in left parahippocampal gyrus, significant differences have been found between heavy smokers and light smokers; **(B)** Heavy smokers showed significant alternations of dFCD measurements in right thalamus compared with nonsmokers, significant differences have been found between heavy smokers and light smokers; **(C)** Light smokers showed significant alternations of dFCD measurements in left middle temporal gyrus (MTG) compared with nonsmokers, significant differences have been found between heavy smokers and light smokers. * means difference between the two groups has statistical significance.

The *post-hoc* results demonstrated that both light and heavy smokers showed decreased dFCD in the brain areas of visual cortex (including bilateral calcarine and right cuneus) and left precuneus, and also showed increased dFCD in right OFC, dorsal striatum (left caudate and right putamen), and left thalamus compared with nonsmokers (Figure 2). In addition, heavy smokers showed an increased dFCD in the right thalamus, while light smokers showed decreased dFCD in MTG in comparison to nonsmokers. Moreover, heavy smokers showed increased dFCD in left MTG and right thalamus, and decreased dFCD in left parahippocampal gyrus compared with light smokers (Figure 3). The details were showed in Supplementary Table S1.

### Correlation Analyses

The results showed that the temporal variability in dFCD in the left MTG was positively correlated with pack-years and FTND (*r* = 0.292, *p* = 0.001, Bonferroni corrected). Temporal variability in the right thalamus was positively correlated with FTND (*r* = 0.265, *p* = 0.003, Bonferroni corrected).

### Validation Analyses

Our results reported above could be validated with different window length of 30 and 80 TRs. The corresponding results are shown in the Supplementary Materials (*p* < 0.01, Supplementary Table S2).

There was no significant difference of mean FD among three groups (*p* = 1.45 for ANOVA). The mean FD in heavy smokers was not significantly different from that in light smokers (*p* = 0.096 for two sample *t*-test). The correlations between mean FD and ROI signals of brain regions were all not significant (*p* > 0.05).

## Discussion

In the current study, we explored altered static and dFCD in chronic smokers compared with nonsmokers. As we assumed, shared abnormalities of dFCD and static FCD both in heavy and light smokers have been found to be possible inherent abnormalities irrelated with the degree of cigarette smoking. In addition, the aberrance of static FCD and dFCD between heavy and light smokers has been found in certain brain areas. Also, the correlation analyses showed that part of the temporal variability in dFCD of different smoking severity was positively correlated with pack-years and FTND, which was originally thought to be associated with the severity of smoking.

In the dFCD study, heavy smokers exhibited significant increases in the left MTG and right thalamus, and a decrease in the left parahippocampal gyrus in comparison to light smokers. As suggested by previous studies, the MTG and parahippocampal gyrus are core components of DMN. Increased dFCD variability in MTG and decreased dFCD variability in parahippocampal gyrus suggests a disturbed integrity of DMN connectivity in the resting state. The DMN is hypothesized to be correlated with internal mentation (30) and is associated with self-referential mental activity and emotional processing tasks (31). To date, the DMN is reported to be implicated in substance use disorders (32), while the chronic nicotine use is reported to negatively impact FC within the DMN, possibly contributing to the difficulty smokers have in quitting (10). Though it is not typically considered to be part of DMN, strong FC has been proved between thalamus and DMN (33). In addition, the variability of dFCD in the right thalamus and left MTG was positively correlated with smoking behaviors, including pack-years and FTND. Given that the localization of significant correlation was primarily in the DMN, the findings were consistent with the incentive-habit model of addiction (34). In chronic smokers, the degree of nicotine dependence is continually reinforced through positive and negative reinforcement, while greater severity is associated with more reliance on habitual use (14). We may draw a conclusion that with the progression of degree of smoking, smoking-related behaviors may become more habitual. Hence, smokers tend to have more difficulty in quitting smoking. Longitudinally, studies could be applied to examine the alternations of functional coordination in brain regions along with the changes of severity of nicotine dependence.

The dFCD study also revealed shared abnormalities of brain regions in right OFC, dorsal striatum (including left caudate and right putamen), left thalamus, and left precuneus in both heavy and light smokers. The frontal-striatal-thalamic circuits is critical for processing of reward (35, 36). Thereinto, the dorsal striatum is associated with motivation, or the drive for action that leads one to work to obtain rewards (37), which drives to obtain smoking-related reward (38). Smoking addicts who are accompanied with dorsal striatum damage were more likely to discontinue smoking. In addition, the characteristics of this interruption is that smoking can be quitted easily and quickly, without recurrence. Furthermore, the impulse to smoke in these people is reduced compared to those smokers without dorsal striatum damage (39). The thalamus is vulnerable to addictive effects of cigarette smoking due to the high density of acetylcholine receptors (AChRs) (40). It participates in the circuit by relaying striatal inputs to the frontal regions and providing feedback to the striatum. Dysregulation of the OFC is correlated with faulty decision-making and the incapacity to inhibit compulsive and repetitive behaviors (41). Imageology studies indicated that the OFC presented hypoactivity during withdrawal in substance use addiction (42, 43). Consistent with previous studies, in this study, the increased dFCD variability in the frontal-striatal-thalamic circuits suggested that regions involved in reward and impulsive-compulsive behavior exhibited more flexibility in functional regulation with other brain networks in smokers. As for precuneus, it is a crucial component part of DMN, which is thought to be associated with self-referential processing such as monitoring craving or withdrawal symptoms (14). Therefore, we hypothesized that the aberrance of the frontal-striatal-thalamic circuits and precuneus might be associated with the craving for nicotine and relapse of smoking behaviors. Furthermore, the above alternations were supposed to be inherent abnormalities related to smoking behaviors.

Compared with nonsmokers, both heavy and light smokers also showed alternations of dFCD in bilateral calcarine and right cuneus. Previous studies have demonstrated that calcarine is a component of the visual attention network and plays a major role in visual information integration and attention processing (44). The cortex around the calcarine fissure is the primary visual cortex (45). The bilateral calcarine are components of the occipital cortex, which is considered as low order brain region in the visual cortex (46). The cuneus is functionally connected to a visual network and is considered to be have crucial role in the integration of visual information (47). According to previous studies, smokers display an initial top-down attention bias toward cigarette cues and demonstrate impairment in inhibiting attentional biases (48, 49). Attentional mechanisms of top-down biasing of feature selection in visual cortex have been extensively investigated, indicating that attention exerts its effect by modulating the gain of neural processing in sensory visual areas (50). Therefore, we supposed that the aberrance in visual cortex in chronic smokers might be associated with the impairment of attention biases.

In the current study, static and dFCD methods illustrated brain regions located in the same areas, including the bilateral calcarine and right cuneus. The reduced dFCD variability in visual cortex in chronic smokers might signify the weakness in neural communication between this area and other regions of the brain, which is consistent with the result of static FCD. Alternations in static FCD suggest the functional impairment in visual network. However, in to dFCD, the static index only showed alternations in the more primitive part of visual cortex. The dynamic index tended to show changes in brain regions that associated with emotion and perception. We supposed that dFCD tends to show fluctuation within a short period of time, and it could provide more subtle variations in brain coordination. A combination of static and dynamic FC has been performed in schizophrenia and depression (20, 51). In addition, we used 30 and 80 TRs with 90% overlap to validate our results. The results showed inconsistent results in some brain regions in different parameters. Hence, we may draw the conclusion that longer or shorter sliding window size might weaken the sensitivity of examining the variance of dynamic changes in brain connectivity. In this study, heavy smokers showed significantly decreasing static FCD in right calcarine and right cuneus. The reason might be that smoking behaviors in smokers with higher degree of nicotine dependence tend to become more habitual. Hence, incentive effects will be weaker, suggesting that heavy smokers' attentional and approach biases for smoking cues should be attenuated compared with light smokers (34). From the above results, we may draw the conclusion that static FCD tends to perform more subtle variations in comparison to dFCD. In addition, the aberrance of static FCD represents impairment of brain FC, while the dFCD demonstrated the alternations of the variance within brain coordination over a short time and supply more subtle information.

The current study still exists several limitations. First, all the subjects in our study are male. Hence, the current study fails to analyze intergender differences. Second, the sample size is small in this study. A larger size of subjects needs to be performed in the future to verify our results. Finally, the current studies are cross-sectional. To investigate the alternations of brain coordination, accompanied with the development of severity of cigarette smoking in chronic smokers, and to elucidate the static and dynamic characteristics of whole-brain connectivity, longitudinal studies are needed to be conducted in future research.

## Conclusion

In conclusion, there exists some brain regions that tend to associate with the severity of nicotine dependence. In addition, chronic smokers showed inherent aberrance which is irrelevant to the severity of nicotine dependence. The dFCD significantly outperforms the static FCD, which could provide more variations. The current findings demonstrated that combing static and dynamic analyses could provide complementary evidence to help people understand the changes of neuroscience in cigarette smokers.

## Data Availability Statement

The original contributions presented in the study are included in the article/Supplementary Material, further inquiries can be directed to the corresponding authors.

## Ethics Statement

The studies involving human participants were reviewed and approved by the Local Medical Ethics Committee of the First Affiliated Hospital of Zhengzhou University. The patients/participants provided their written informed consent to participate in this study.

## Author Contributions

ZY contributed to the experiments, data analysis and writing of the manuscript. MW, YW, and HH contributed to performing the experiments and writing and revising the manuscript. WW, XG, and MZ contributed to the data collection. RZ revised the manuscript. YZ, JC, and SH are the guarantor of this study and had complete access to all data in the study. All authors contributed to the article and approved the submitted version.

## Funding

This research study was supported by the Natural Science Foundation of China (Nov. 81871327).

## Conflict of Interest

The authors declare that the research was conducted in the absence of any commercial or financial relationships that could be construed as a potential conflict of interest.

## Publisher's Note

All claims expressed in this article are solely those of the authors and do not necessarily represent those of their affiliated organizations, or those of the publisher, the editors and the reviewers. Any product that may be evaluated in this article, or claim that may be made by its manufacturer, is not guaranteed or endorsed by the publisher.
