# Supplementary material for: Alternations in Dynamic and Static Functional Connectivity Density in Chronic Smokers
Source: Front Psychiatry. 2022 Apr 21;13:843254. doi: 10.3389/fpsyt.2022.843254 (PMC9068985; doi:10.3389/fpsyt.2022.843254)
Supplement: Supplementary file 1 [file Data_Sheet_1.pdf]

Table S1: Results of post hoc analysis, all p values were original p values.

Regions	HS> HC (t, p)	LS >HC (t, p)	HS > LS (t, p)
R.OFC	3.641, <0.001	5.167, <0.001	-1.937, 0.055
L.CAU	5.120, <0.001	5.179, <0.001	-0.673, 0.502
R.PUT	3.507, 0.001	5.235, <0.001	-1.919, 0.058
L.THA	4.169, <0.001	4.815, <0.001	-0.911, 0.364
L.PHG	2.676, 0.009	5.127, <0.001	-3.111, 0.002
R.CUN	-3.920, <0.001	-3.343, 0.001	-0.481, 0.631
R.CAL	-3.251, 0.002	-3.621, <0.001	0.634, 0.527
L.CAL	-3.390, 0.001	-2.799, <0.001	-1.029, 0.306
L.MTG	-0.568, 0.571	-4.418, <0.001	3.563, 0.001
L.PCUN	-2.744, 0.007	-3.844, <0.001	1.925, 0.057
R.THA	3.381, 0.001	0.455, 0.650	3.258, 0.001

Note: HS: heavy smokers; LS: light smokers; HC: healthy controls; R.OFC: right orbitofrontal cortex; L.CAU: left caudate; R.PUT: right putamen; L.THA: left thalamus; L.PHG: left parahippocampal gyrus; R.CUN: right cuneus; R.CAL: right calcarine; L.CAL: left calcarine; L.MTG: left middle temporal gyrus; L.PCUN: left precuneus; R.THA: right thalamus

Table S2: Validated results with a window length of 30 TRs and 80 TRs.

Indices	Regions	P for ANOVA
30TRs	R.THA	<0.001
	L.THA	<0.001
	L.MTG	<0.001
	L.CAL	0.001
	L.PCUN	0.001
80TRs	L.THA	<0.001
	R.THA	0.001
	L.CAL	0.002
	L.PUT	0.002
	L.CUN	0.002

Note: Significant differences still existed with different window length in main brain areas ($p < 0.01$, uncorrected) ; Abbreviations: L, left; R, right; R.THA, right thalamus; L.THA, left thalamus; L.MTG, left middle temporal gyrus; L.CAL, left calcarine; L.PCUN, left precuneus; L.PUT, left putamen.
